# Intracorporeal knotting of a femoral nerve catheter

**DOI:** 10.3205/iprs000063

**Published:** 2015-01-28

**Authors:** Mohamed Ghanem, Jörg Schnoor, Martin Wiegel, Christoph Josten, Andreas W. Reske

**Affiliations:** 1 Department of Orthopedic, Trauma and Plastic Surgery, University Hospital Leipzig, Leipzig, Germany; 2 Department of Anesthesiology and Intensive Care Medicine, University Hospital Leipzig, Leipzig, Germany; 3 ACQUA Clinic, Leipzig, Germany

**Keywords:** postoperative pain management, regional anesthesia, peripheral nerve catheters, femoral nerve catheter, complications

## Abstract

Peripheral nerve catheters are effective and well-established tools to provide postoperative analgesia to patients undergoing orthopedic surgery. The performance of these techniques is usually considered safe. However, placement of nerve catheters may be associated with a considerable number of side effects and major complications have repeatedly been published. In this work, we report on a patient who underwent total knee replacement with spinal anesthesia and preoperative insertion of femoral and sciatic nerve catheters for postoperative analgesia. During insertion of the femoral catheter, significant resistance was encountered upon retracting the catheter. This occurred due to knotting of the catheter. The catheter had to be removed by operative intervention which has to be considered a major complication. The postoperative course was uneventful. The principles for removal of entrapped peripheral catheters are not well established, may differ from those for neuroaxial catheters, and range from cautious manipulation up to surgical intervention.

## Zusammenfassung

Periphere Nervenkatheter sind etablierte Verfahren zur postoperativen Analgesie bei Patienten mit größeren orthopädischen Eingriffen. Die Effektivität dieser Techniken gilt in der Regel als sicher. Jedoch kann die Platzierung von Nervenkathetern mit einer beträchtlichen Anzahl von Nebenwirkungen und schweren Komplikationen assoziiert sein. In dieser Arbeit berichten wir über einen Patienten, der mit einem Kniegelenksersatz versorgt wurde. Neben der Spinalanästhesie erfolgte die Anlage von Ischiasnerv- sowie Femoralisnervkatheter zur postoperativen Analgesie. Beim Einbringen des femoralen Nervkatheters ist ein eindeutiger Widerstand beim Zurückziehen des Katheters aufgetreten. Dies geschah durch Knotenbildung des Katheters. Der Katheter musste durch einen operativen Eingriff entfernt werden, dies gilt damit als schwere Komplikation. Der postoperative Verlauf war allerdings komplikationslos. Die Grundprinzipien für die Entfernung von eingeschlossenen peripheren Nervkatheter sind noch nicht gut etabliert, unterscheiden sich von denen für neuroaxiale Katheter und reichen von vorsichtiger Manipulation bis hin zu chirurgischem Eingriff.

## Introduction

An effective option to provide postoperative analgesia to patients undergoing surgery of the knee is the insertion of femoral nerve catheters [1], [2], [3]. Although major complications of these techniques seem to be rare, their increasing use may lead to the occurrence of rare problems [4], [5], [6]. Reports on such events provide important information to all practitioners of regional anesthesia. Our aim is to report on the complication we encountered in order to contribute to the recognition of potential risks and, possibly, to improve the techniques involved.

## Case presentation

The patient consented to publication of this report.

**Figure 1:**
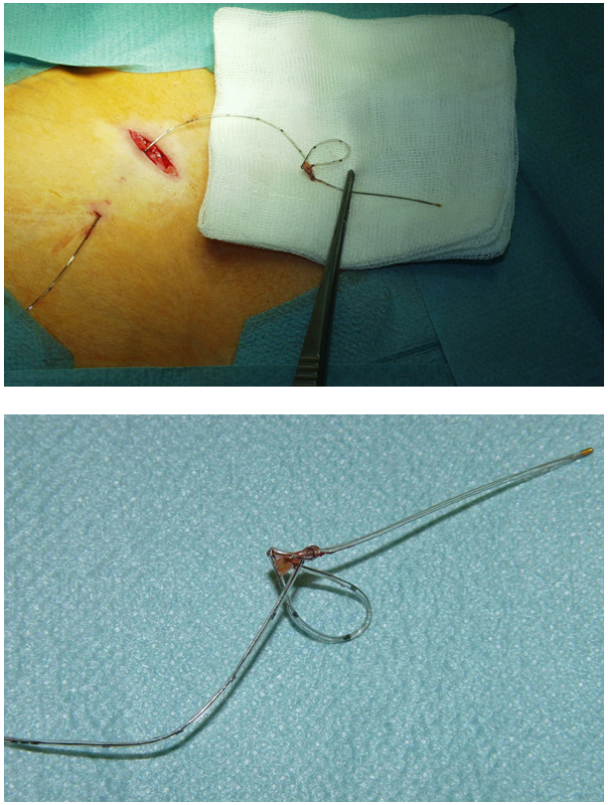
Upper panel – situation after removal of the knotted catheter. Lower panel – the knotted catheter in greater detail, marks on the catheter are one cm apart.

A 42-yr-old ASA II woman with bilateral gonarthrosis was scheduled for left-sided total knee arthroplasty. Spinal anesthesia and preoperative insertion of femoral and sciatic nerve catheters for postoperative analgesia were planned. Following Winnie’s approach, the femoral nerve was located without problems in a depth of 4 cm using a nerve stimulator (HNS 11^TM^, Braun, Melsungen, Germany) [7]. No unusual resistance was felt during needle insertion. Twenty milliliters of 0.2% ropivacaine were injected and the catheter (Stimulong Plus^TM^, Pajunk, Geisingen, Germany) was inserted. The catheter could be threaded very easily and was inadvertently inserted to a depth of 25 cm (we usually thread femoral nerve catheters 5–8 cm beyond the needle tip) [7]. It was tunneled subcutaneously and then retracted without problems until, at the 15-cm-mark, significant resistance was encountered. Traction was applied until the catheter began to stretch so that the distance between two 1-cm markings enlarged significantly. After having consulted two colleagues proficient in regional anesthesia as well as the orthopedic surgeon, we decided to remove the catheter by operative intervention. The patient was informed about the problem and she consented to the surgical removal. Insertion of the sciatic nerve catheter, and performance of spinal anesthesia were performed without problems [7]. Surgical dissection of the tissues at the catheter insertion site showed that the catheter had passed through the ileopectineal arch and formed a knot dorsal to it. After removal of the intact catheter, inspection revealed a true knot six centimeters away from the catheter tip (Figure 1 (Fig. 1)). The total knee arthroplasty as well as the postoperative course of the patient were uncomplicated. 

## Discussion

Peripheral nerve catheters are effective options to provide postoperative analgesia to patients undergoing orthopedic surgery and the performance of these techniques is usually considered safe. However, placement of nerve catheters may be associated with a considerable number of side effects and major complications have repeatedly been published [6]. Reports on complications help to recognize potential risks and, possibly, to improve the techniques involved. Although the patient’s postoperative course was uneventful, the knotted catheter had to be removed by operative intervention which has to be considered a major complication.

In total, the knotting of peripheral nerve catheters is a relatively uncommon phenomenon, occurring in 0.13% of patients [8], [9], [10]. In our opinion, two aspects warrant further discussion, i.e. how to avoid the knotting of peripheral nerve catheters, and what to do if it occurs. 

Knotting a catheter might result from coiling due to threading the catheter too far. This notion is supported by two other reports describing knotted femoral and fascia iliaca catheters. In both reports, catheters were threaded 10–20 cm beyond the needle tip, and coiling of the catheter was considered the underlying mechanism [1], [11]. Although a greater insertion length might be associated with a higher incidence of coiling of the catheter, coiling does not necessarily lead to knots and, shorter insertion does not exclude knotting of the catheter [12]. In consequence, different insertion lengths of femoral nerve catheters have been reported, but the optimal insertion length still remains unknown [13], [14], [15]. Although there is not enough information available to establish a direct relationship between insertion length and catheter knotting, it seems prudent that coiling and knotting a catheter requires a corresponding length of the catheter in situ. Considering the multiple catheter knots reported with insertion beyond 5 cm and the lack of data suggesting that insertion lengths above 5 cm are beneficial, recommending a maximal insertion length of 5 cm seems well justified [6].

The removal of a knotted peripheral catheter should first be tried by altering the patient’s position to minimize pressure and tension on the perineural soft tissues according to the recommendations of Offerdahl and colleagues [1]. There, it appears difficult to define the amount of traction that can be applied safely. Not only will it vary among different catheter types, but also will the anatomical structure differ within which the knot is trapped. In our patient, the knot was wedged within the ileopectineal arch and, most likely, the catheter might have broken if we had applied forced traction. If the knot is located in the vicinity of or around a nerve, applying traction may have catastrophic sequelae.

We found that the principles for removal of entrapped peripheral catheters are not well known and may differ from those for neuraxial catheters [1]. If a knotted peripheral catheter could not be manually removed, the use of mobile ultrasound machines or radiological imaging may help to localize the perineural catheter in order to facilitate decision-making. In our case, we were highly concerned about the stretching of the catheter when traction was applied. We felt that the risk of catheter breakage and anatomical damage justified its surgical removal, particularly, in face of the planned surgery. This decision was guided by recommendations for removal of superficial catheters, whilst deeper catheters without clinical symptoms i.e. epidural catheters could be left in situ. In our context, early surgical removal was performed also because bacterial colonization of the femoral catheter might cause infection. 

## Conclusion

We conclude that knotting of a nerve catheter might result from threading the catheter too far. How far to insert a peripheral catheter still remains unknown, but minimizing the insertion length of catheters to 5 cm seems prudent. The principles for removal of entrapped peripheral catheters are not well established and may differ from those for neuroaxial catheters. Techniques for catheter removal range from cautious manipulation up to surgical intervention. Patient re-positioning may facilitate manual catheter removal and thus be an option before surgical intervention is indicated.

## Notes

### Competing interests

The authors declare that they have no competing interests.

## References

[1] Offerdahl MR, Lennon RL, Horlocker TT (2004). Successful removal of a knotted fascia iliaca catheter: principles of patient positioning for peripheral nerve catheter extraction. Anesth Analg.

[2] Capdevila X, Barthelet Y, Biboulet P, Ryckwaert Y, Rubenovitch J, d'Athis F (1999). Effects of perioperative analgesic technique on the surgical outcome and duration of rehabilitation after major knee surgery. Anesthesiology.

[3] Singelyn FJ, Deyaert M, Joris D, Pendeville E, Gouverneur JM (1998). Effects of intravenous patient-controlled analgesia with morphine, continuous epidural analgesia, and continuous three-in-one block on postoperative pain and knee rehabilitation after unilateral total knee arthroplasty. Anesth Analg.

[4] Capdevila X, Pirat P, Bringuier S, Gaertner E, Singelyn F, Bernard N, Choquet O, Bouaziz H, Bonnet F, French Study Group on Continuous Peripheral Nerve Blocks (2005). Continuous peripheral nerve blocks in hospital wards after orthopedic surgery: a multicenter prospective analysis of the quality of postoperative analgesia and complications in 1,416 patients. Anesthesiology.

[5] Wiegel M, Gottschaldt U, Hennebach R, Hirschberg T, Reske A (2007). Complications and adverse effects associated with continuous peripheral nerve blocks in orthopedic patients. Anesth Analg.

[6] Ilfeld BM (2011). Continuous peripheral nerve blocks: a review of the published evidence. Anesth Analg.

[7] Winnie AP, Ramamurthy S, Durrani Z (1973). The inguinal paravascular technic of lumbar plexus anesthesia: the “3-in-1 block”. Anesth Analg.

[8] Hübner T, Gerber H (2003). Der Knoten im axillären Plexus-brachialis-Katheter. Eine seltene Komplikation. Anaesthesist.

[9] Rudd K, Hall PJ (2004). Knotted femoral nerve catheter. Anaesth Intensive Care.

[10] Burgher AH, Hebl JR (2007). Minimally invasive retrieval of knotted nonstimulating peripheral nerve catheters. Reg Anesth Pain Med.

[11] Motamed C, Bouaziz H, Mercier FJ, Benhamou D (1997). Knotting of a femoral catheter. Reg Anesth.

[12] Tran QD, Gordon A, Asenjo JF, de la Cuadra-Fontaine JC (2005). Retained and cut stimulating infraclavicular catheter. Can J Anaesth.

[13] Ganapathy S, Wasserman RA, Watson JT, Bennett J, Armstrong KP, Stockall CA, Chess DG, MacDonald C (1999). Modified continuous femoral three-in-one block for postoperative pain after total knee arthroplasty. Anesth Analg.

[14] Capdevila X, Biboulet P, Morau D, Bernard N, Deschodt J, Lopez S, d'Athis F (2002). Continuous three-in-one block for postoperative pain after lower limb orthopedic surgery: where do the catheters go?. Anesth Analg.

[15] Boezaart AP (2006). Perineural infusion of local anesthetics. Anesthesiology.

